# Supplementary material for: Mössbauer spectroscopy of a monolayer of single molecule magnets
Source: Nat Commun. 2018 Feb 2;9:480. doi: 10.1038/s41467-018-02840-w (PMC5797240; doi:10.1038/s41467-018-02840-w)
Supplement: Supplementary file 1 — Supplementary Information [file 41467_2018_2840_MOESM1_ESM.pdf]

SUPPLEMENTARY FIGURES

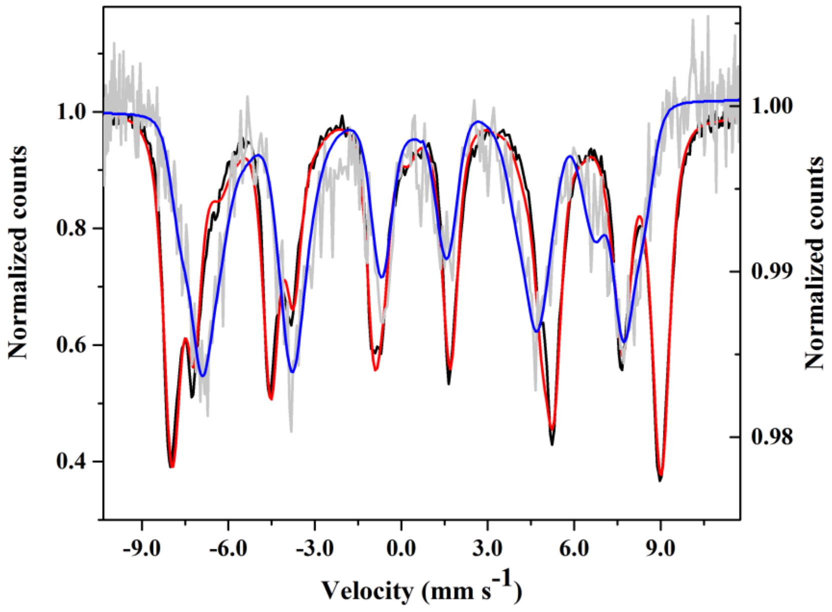

Supplementary Figure 1. *Comparison between the Mössbauer spectrum of the dropcast sample at 2.2 K (left scale, measured spectrum in black and relative fit in red) and the Mössbauer spectrum of the monolayer sample at 2.2 K (right scale, measured spectrum in gray and relative fit in blue).*

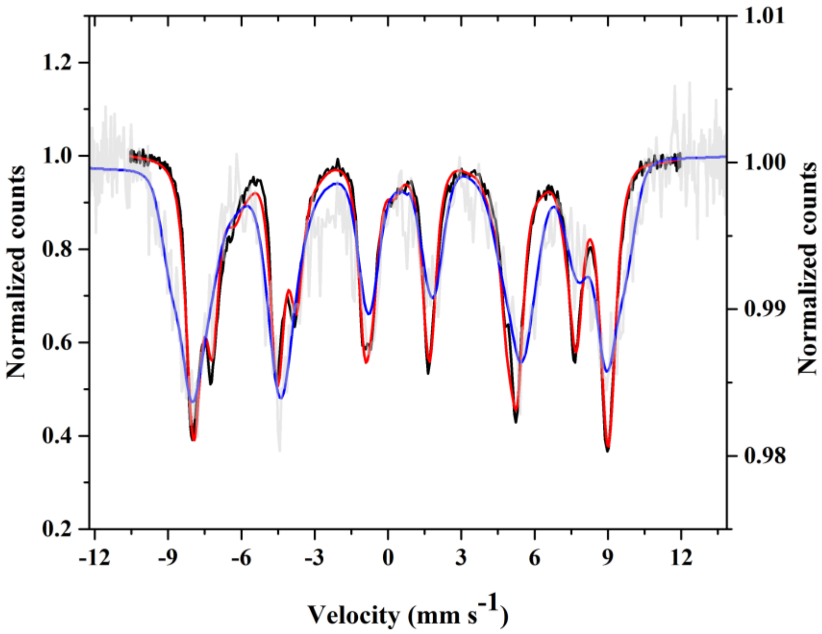

Supplementary Figure 2. *Comparison between the Mössbauer spectrum of the dropcast sample at 2.2 K (left scale, measured spectrum in black and relative fit in red) and the Mössbauer spectrum of the monolayer sample at 2.2 K (right scale, measured spectrum in gray and relative fit in blue). The x axis of the monolayer spectrum has been scaled with respect to that of the dropcast sample.*

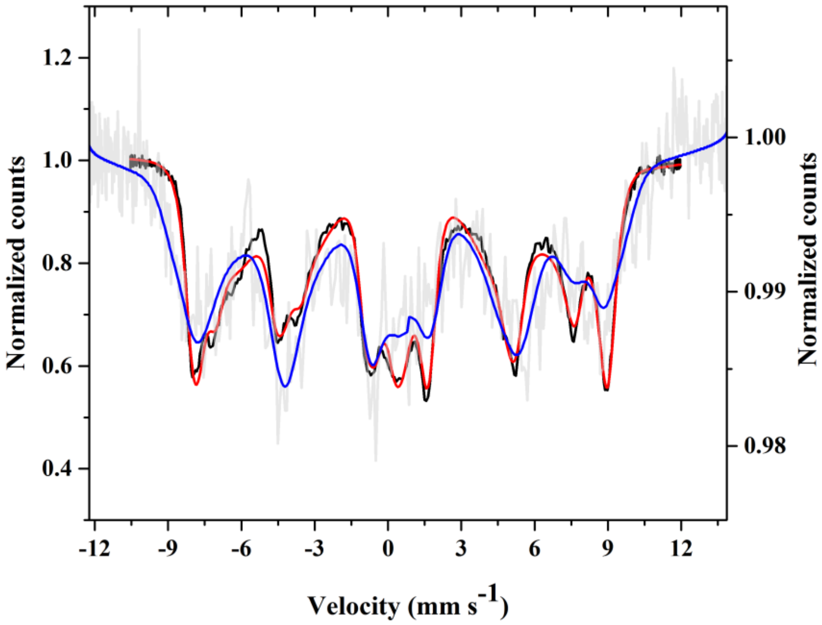

Supplementary Figure 3. *Comparison between the Mössbauer spectrum of the dropcast sample at 7.0 K (left scale, measured spectrum in black and relative fit in red) and the Mössbauer spectrum of the monolayer sample at 7.0 K (right scale, measured spectrum in gray and relative fit in blue). The x axis of the monolayer spectrum has been scaled with respect to that of the dropcast sample.*

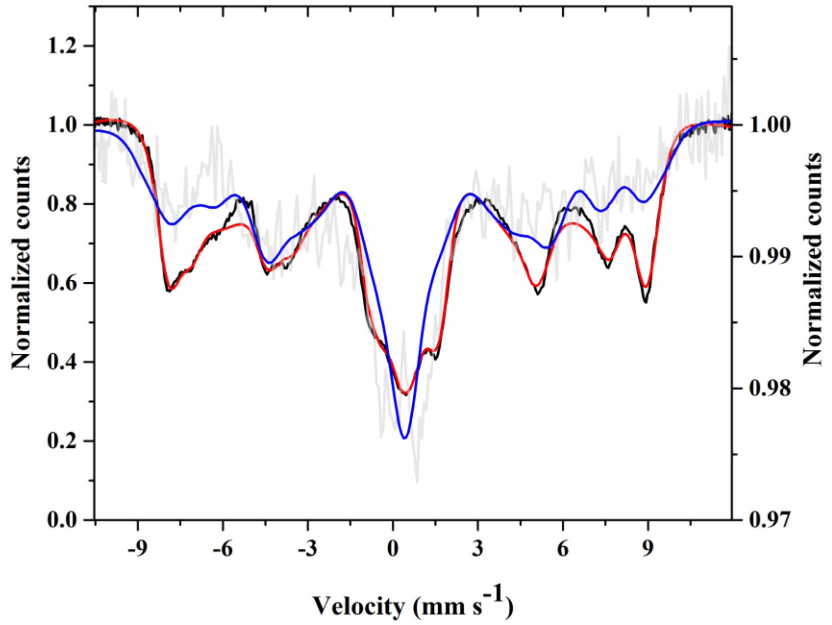

Supplementary Figure 4. *Comparison between the Mössbauer spectrum of the dropcast sample at 11 K (left scale, measured spectrum in black and relative fit in red) and the Mössbauer spectrum of the monolayer sample at 11 K (right scale, measured spectrum in gray and relative fit in blue). The x axis of the monolayer spectrum has been scaled with respect to that of the dropcast sample.*

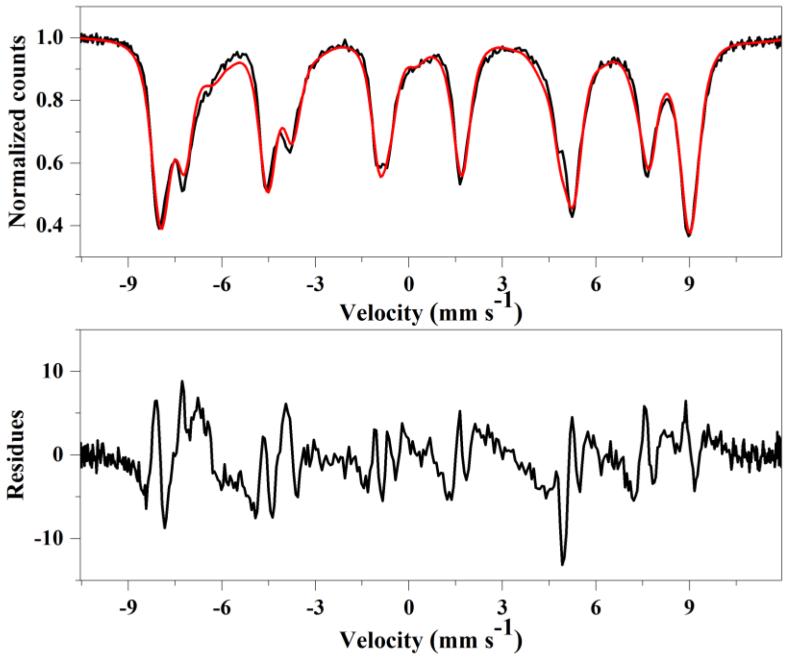

Supplementary Figure 5. *Top panel: Mössbauer spectrum of the dropcast sample at 2.2 K (measured spectrum in black and relative fit in red). Bottom panel: residuals of the fitting procedure.*

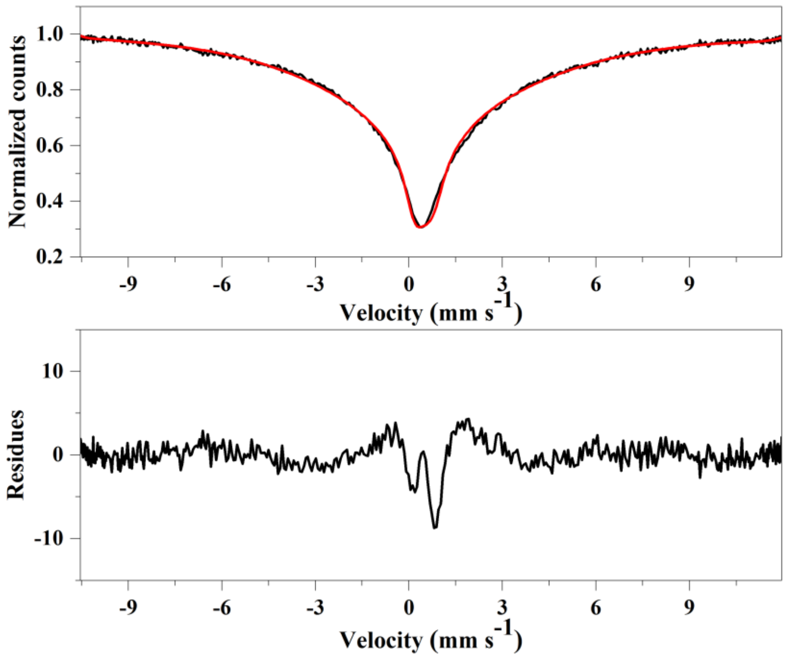

Supplementary Figure 6. *Top panel: Mössbauer spectrum of the dropcast sample at 40 K (measured spectrum in black and relative fit in red). Bottom panel: residuals of the fitting procedure.*

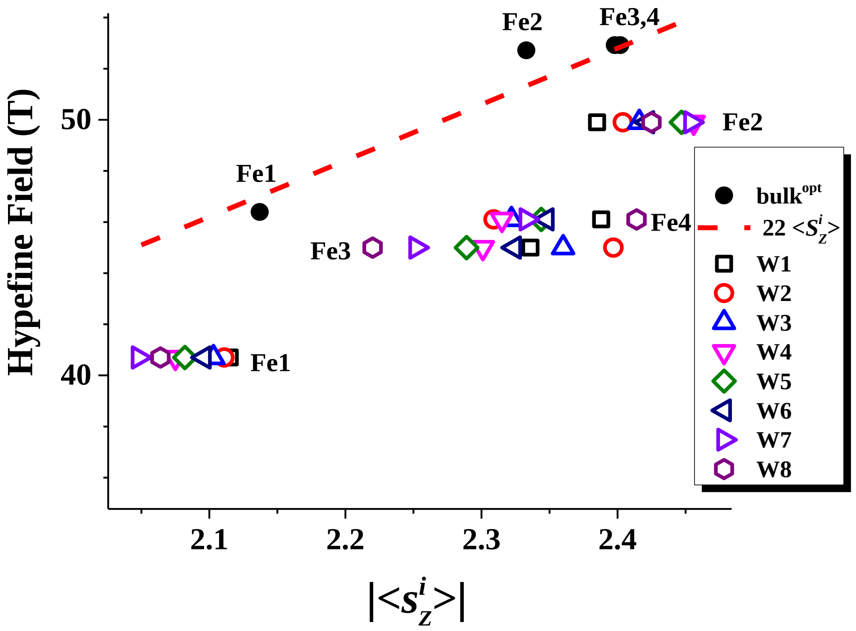

Supplementary Figure 7. *Magnetic hyperfine fields extracted from the fit of the dropcast (solid black dots) and the monolayer (empty symbols) spectra at 2.2 K reported as a function of the local spin components* $|\langle s_z^i \rangle|$ *evaluated for the optimized structure in the bulk phase and for each walker in AIMD calculations (see legend). The red broken line represents the behaviour expected from the* $22\langle s_z \rangle$ *rule for the Fermi contact field* [13].

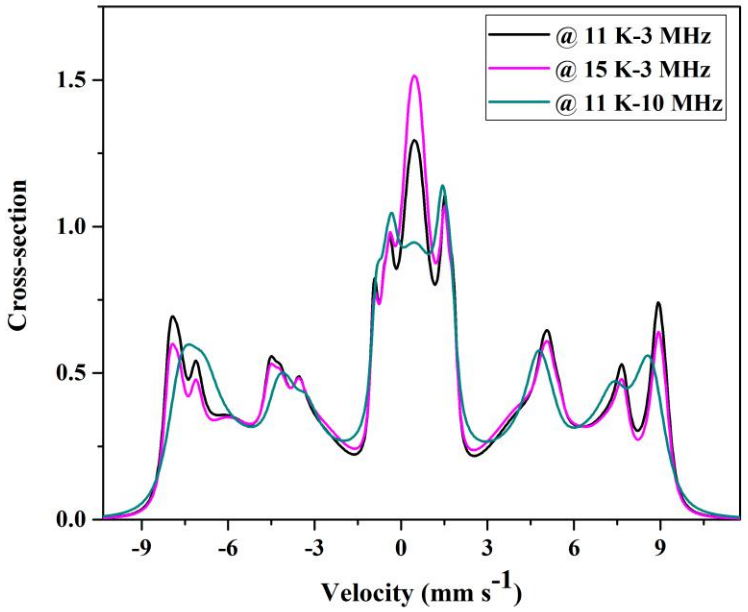

Supplementary Figure 8. *Absorption cross-section obtained by fitting the spectrum of the dropcast sample at 11 K (the transition rate extracted from the fit is 3 MHz) (black line), cross-section simulated for T = 15 K (or a 27-% reduced |D| value) while leaving the other parameters unchanged (magenta line), and cross-section simulated at T = 11 K for a higher transition rate (10 MHz), expected to occur at 15 K (cyan line).*

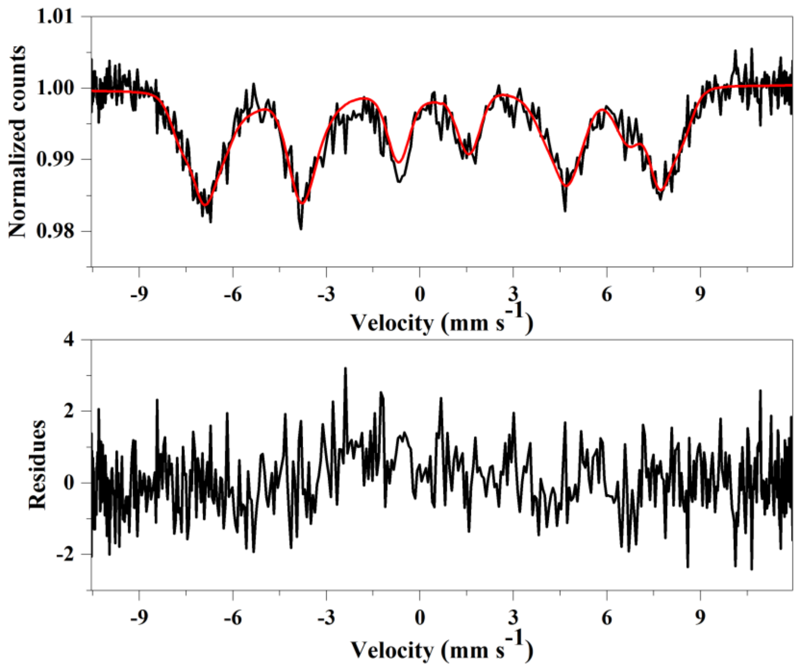

Supplementary Figure 9. *Top panel: Mössbauer spectrum of the monolayer sample at 2.2 K (measured spectrum in black and relative fit in red). Bottom panel: residuals of the fitting procedure.*

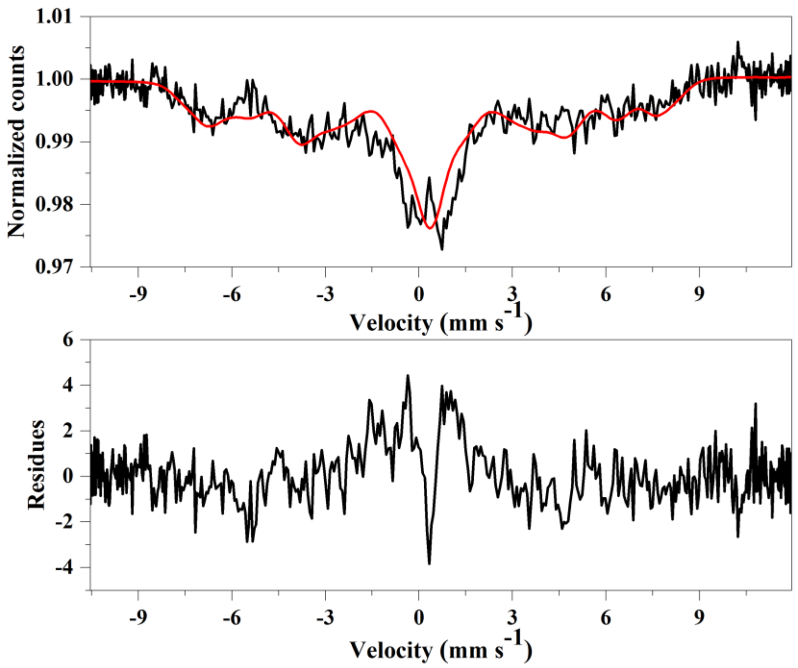

Supplementary Figure 10. *Top panel: Mössbauer spectrum of the monolayer sample at 11 K (measured spectrum in black and relative fit in red). Bottom panel: residuals of the fitting procedure.*

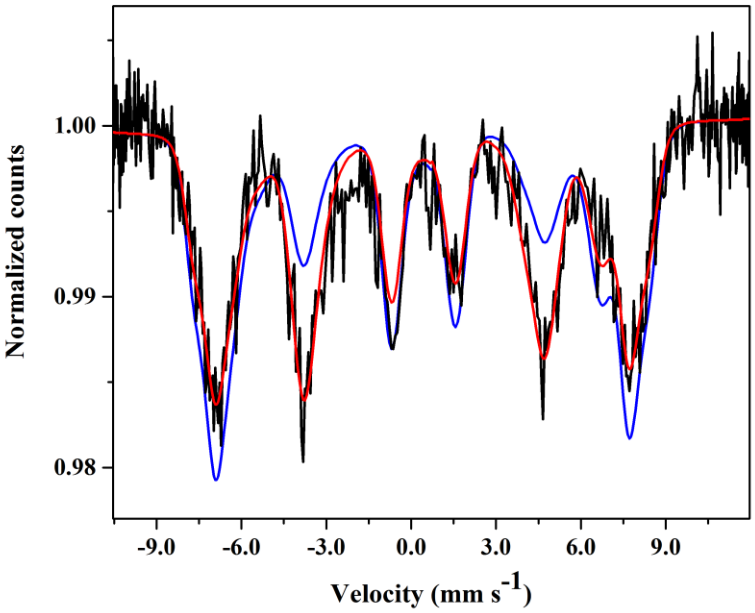

Supplementary Figure 11. *Mössbauer spectrum of the monolayer sample at 2.2 K (black line) and corresponding fit assuming a random orientation of molecules on the substrate (red line). The blue line is a simulation of the same system imposing a preferential orientation of the molecules as in ref.* [4].

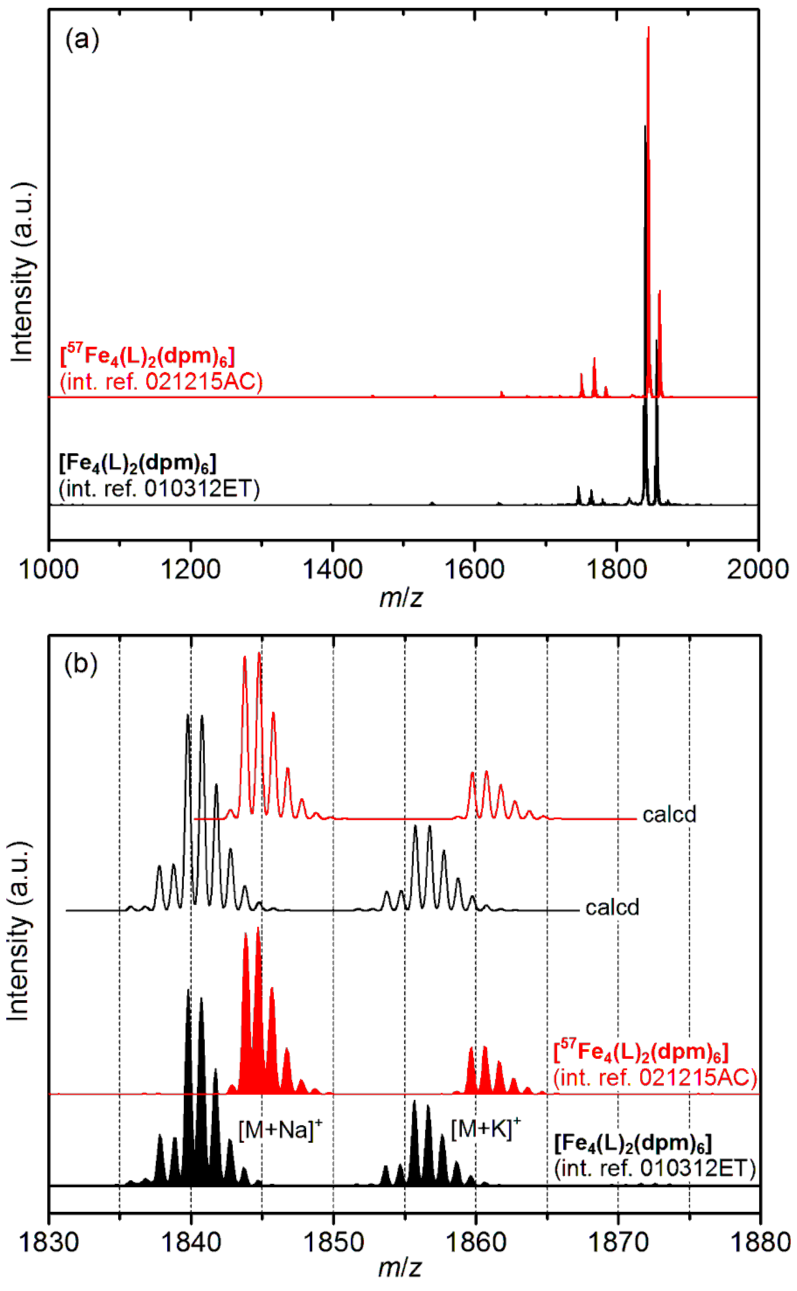

Supplementary Figure 12. *Full-range (a) and mass scale-expanded (b) ESI-MS spectra of [^{57}Fe$_4$(L)$_2$(dpm)$_6$] (red) and [Fe$_4$(L)$_2$(dpm)$_6$] [4] (black) in 2-propanol:CH_2Cl_2 3:1 v/v (~0.5 mg/mL). The calculated isotopic patterns [14] are also presented in (b) using the same color code. The calculated pattern for the ^{57}Fe-enriched complex is based on the following isotopic composition for iron: ^{54}Fe (0.0%), ^{56}Fe (1.4%), ^{57}Fe (97.2%) and ^{58}Fe (1.4%).*

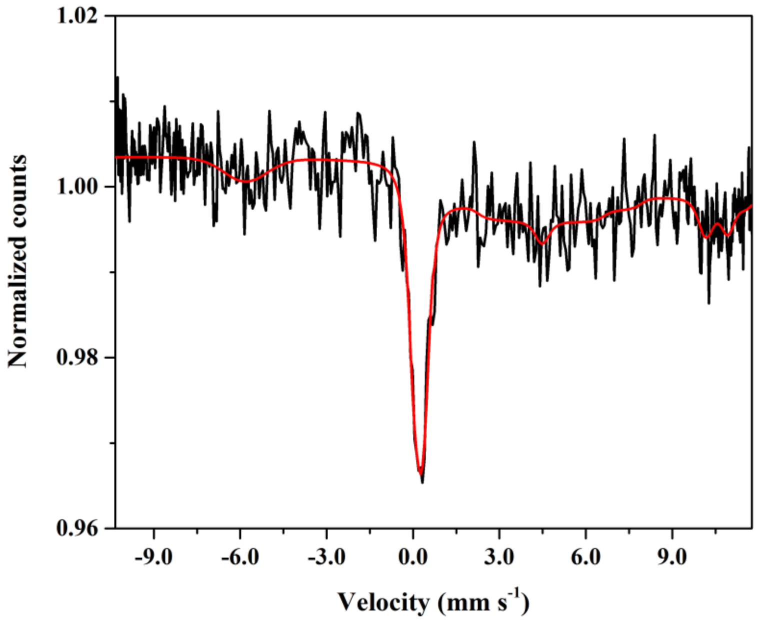

Supplementary Figure 13. *"Empty-can" Mössbauer spectrum of the beamline (black line) and relative fit (red line).*

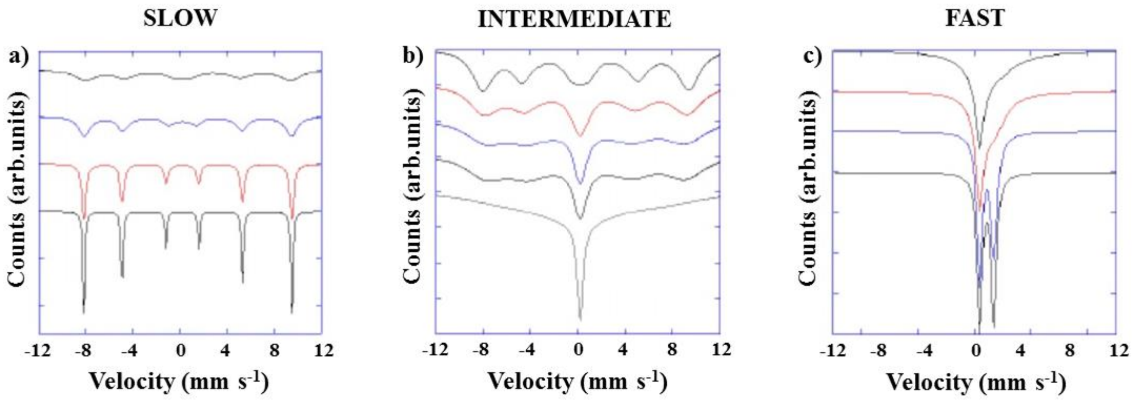

Supplementary Figure 14. *Simulated Mössbauer spectra of the slow (a), intermediate (b) and fast (c) dynamic regimes, respectively*[8]*. In panel (a), the R value increases from bottom to top, while in panels (b) and (c) from top to bottom.*

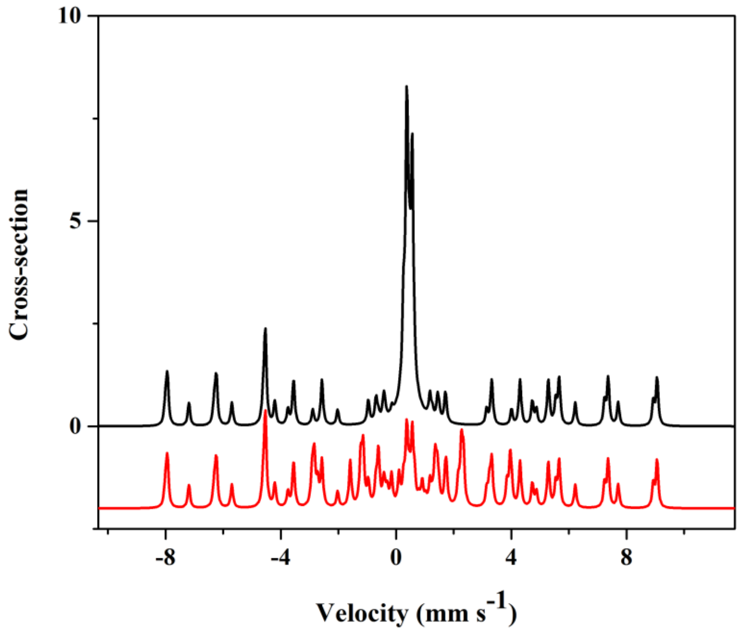

Supplementary Figure 15. *Simulations of the high temperature cross-sections ignoring the interaction with the thermal bath for two values of transverse anisotropy parameter (red line: E = 0; black line: E = 0.003 K).*

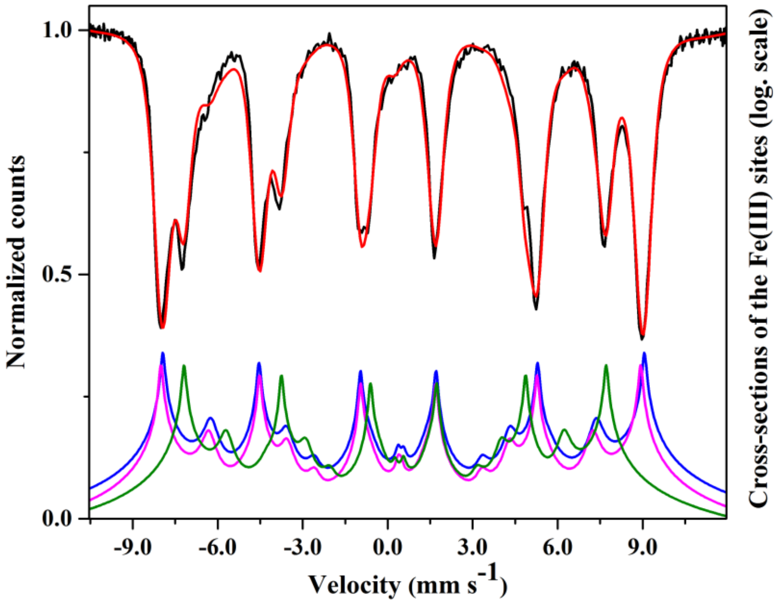

Supplementary Figure 16. *Mössbauer spectra of Figure 3a in the manuscript with the cross-sections of the three contributions reported in logarithmic scale.*

SUPPLEMENTARY TABLES

Supplementary Table 1. *Isomer shift with respect to α-Fe (mm s^{-1}) for the dropcast sample*

Temperature (K)	Central site	Peripheral sites	
2.2	0.409(2)	0.418(4)	0.467(2)
2.9	0.405(2)	0.44(1)	0.469(5)
5.0	0.442(3)	0.528(6)	0.425(3)
7.0	0.469(5)	0.584(6)	0.410(4)
11	0.487(6)	0.682(4)	0.369(3)
17	0.479(9)	0.56(1)	0.429(6)
20	0.49(2)	0.57(1)	0.406(8)
24	0.49(2)	0.63(1)	0.382(7)
32	0.46(2)	0.684(5)	0.467(7)
40	0.62(2)	0.701(2)	0.280(6)

Last column refers to signals with double intensity attributed to two undistinguishable peripheral sites.

Supplementary Table 2. *Electric quadrupole shift (mm s^{-1}) for the dropcast sample*

Temperature (K)	Central site	Peripheral sites	
2.2	-0.149(2)	0.045(4)	0.094(2)
2.9	-0.138(2)	0.048(5)	0.071(2)
5.0	-0.163(3)	0.030(3)	0.086(1)
7.0	-0.166(5)	0.010(1)	0.110(2)
11	-0.199(6)	0.042(4)	0.079(2)
17	-0.28(1)	0.012(3)	0.130(5)
20	-0.30(2)	0.012	0.135(5)
24	-0.23(2)	0.012	0.125(5)
32	-0.22(3)	0.012	0.14(1)
40	-0.21(1)	0.12(2)	0.085(7)

Last column refers to signals with double intensity attributed to two undistinguishable peripheral sites. Parameters without error are held fixed in the fitting procedure.

Supplementary Table 3. *Absolute value of the hyperfine magnetic field (in T) generated at the different iron sites by the total spin states* $M_S=\pm 5$ *in the dropcast sample*

Temperature (K)	Central site	Peripheral sites	
2.2	46.39(1)	52.72(2)	52.92(2)
2.9	46.30(2)	52.55(7)	52.88(3)
5.0	46.31(2)	52.92(5)	52.68(3)
7.0	46.16(4)	52.1(1)	53.23(3)
11	46.58(5)	53.0(7)	53.16(3)
17	48.1(1)	54.4(4)	54.4(2)
20	47.9(2)	54.8(5)	54.5(3)
24	47.7(3)	55.5(5)	54.6(2)
32	47.5(7)	55(1)	53.2(7)
40	46.4	52.7	52.9

Last column refers to signals with double intensity attributed to two undistinguishable peripheral sites. Parameters without error are held fixed in the fitting procedure.

Supplementary Table 4. *Fe-O distances (in Å) within the magnetic core of Fe_4 molecules chemically grafted to the Au(111) surface and in the bulk phase*

		$\langle d \rangle_{mono}^{(a)}$	σ	$d_{bulk}^{opt(b)}$
Fe1	**O1**	1.960	0.020	1.991
Fe1	**O2**	2.043	0.046	1.991
Fe1	**O4**	1.965	0.034	1.968
Fe1	**O6**	1.982	0.027	1.984
Fe1	**O7**	1.983	0.031	1.984
Fe1	**O9**	1.969	0.016	2.004
Fe2	**O1**	2.045	0.027	1.979
Fe2	**O2**	1.979	0.028	1.988
Fe2	O13	2.026	0.022	2.034
Fe2	O14	2.033	0.023	2.044
Fe2	O15	2.050	0.028	2.071
Fe2	O16	2.008	0.011	2.058
Fe3	O5	2.010	0.021	2.021
Fe3	**O6**	2.057	0.042	2.013
Fe3	**O9**	1.987	0.023	2.037
Fe3	O10	1.976	0.016	2.020
Fe3	O11	2.088	0.032	2.060
Fe3	O12	2.020	0.021	2.015
Fe4	**O4**	2.005	0.026	2.004
Fe4	**O7**	2.028	0.024	1.979
Fe4	O8	1.998	0.021	2.042
Fe4	O17	2.052	0.027	1.997
Fe4	O18	2.001	0.014	2.027
Fe4	O19	2.013	0.019	2.110

The distances were estimated through AIMD calculations, as described in ref. [12]. Oxygen atoms in bold are those of the tripodal ligands responsible for super-exchange magnetic interactions leading to the ground $S = 5$ state.

a) Distances for the surface-grafted molecule, averaged over 8 distinct trajectories (walkers) of the AIMD simulation; the corresponding standard deviations (σ) are also provided.
b) Distances in the bulk phase obtained after structure optimization by *ab initio* calculations; experimental distances were not included to allow a better comparison with on-surface geometry.

Supplementary Table 5. *Magnetic anisotropy parameters for the single iron sites and for the S = 5 ground state, and intramolecular exchange interactions*

	Bulkopt [a]	W@1-8[b]	W@1-8[c]	W1@[d]	W1@[e]	W3@[d]	W3@[e]
D_1	-1.51	-1.1 (1.3)	-	2.24	-	-1.5	-
D_2	0.92	0.81 (0.08)	-	0.94	-	0.82	-
D_3	0.89	-0.4 (0.4)	-	-0.54	-	-0.58	-
D_4	0.90	0.6 (0.5)	-	0.66	-	0.74	-
$D_{S=5}$	-0.61	-0.58 (0.10)	-	-0.56	-	-0.84	-
J_{12}	23.3	24 (4)	24 (4)	30.9	29.9	24.0	24.9
J_{13}	16.8	47 (12)	44 (12)	40.1	37.7	36.1	35.0
J_{14}	16.4	40 (9)	38 (7)	30.1	29.1	43.4	41.3

Anisotropy parameters, *D*, and exchange interactions, *J*, are given in K and are estimated through *ab initio* calculations as extracted from ref. [12].

a) *D* and *J* values for the bulk phase obtained on optimized geometries by *ab initio* calculations with PBE and PBE+U functionals, respectively; the experimental geometry was not used to allow a better comparison with values obtained on optimized on-surface geometries.
b) *D* and *J* values for the molecule extrapolated from the substrate (@) are calculated with PBE and PBE+U functional, respectively, and are averaged over 8 distinct trajectories (walkers) of the AIMD simulation; the corresponding standard deviations are also provided.
c) Same as (b) but including the Au substrate and calculated with PBE+U functional. In this scenario, the calculation of the *D* values becomes unaffordable due to the size of the simulation cell.
d) Same as (b) but for the indicated individual walker.
e) Same as (c) but for the indicated individual walker.

Supplementary Table 6. *Averaged Mulliken spin densities*

	**Fe1**	**Fe2**	**Fe3**	**Fe4**
Bulk$^{opt\ a}$	4.12	4.19	4.19	4.19
< *Wi@Au*>	3.93	4.12	4.10	4.10
<*Wi~~@~~Au*>	4.09	4.15	4.15	4.16
ΔA_{iso} ^{b} < *Wi@Au*>	-2.11	-0.72	-0.98	-0.98
ΔA_{iso} ^{b} <*Wi~~@~~Au*>	-0.34	-0.44	-0.43	-0.38

Absolute values of the total Mulliken spin density at the four iron sites are computed for the crystalline phase and for the molecule adsorbed on the gold surface including (@) and excluding (~~@~~) the electronic effect of the latter. The values are averaged over the 8 AIMD walkers [12].

a) Computed over one of the four Fe_4 molecules (while keeping the other three fixed) present in the optimized crystal cell [12] with the pseudopotential-GPW approach and periodic boundary conditions (CP2K).

b) Hyperfine field variation (in T) induced by the spin density variation upon grafting on gold surface computed with the pseudopotential-GPW approach and periodic boundary conditions (CP2K).

Supplementary Table 7. *Calculated Mulliken spin densities for individual walkers*

	**Fe1**	**Fe2**	**Fe3**	**Fe4**
W1@Au	3.94	4.10	4.12	4.12
W1~~@~~Au	4.08	4.15	4.16	4.15
W2@Au	3.98	4.13	4.10	4.09
W2~~@~~Au	4.10	4.15	4.16	4.16
W3@Au	3.88	4.13	4.13	4.14
W3~~@~~Au	4.09	4.16	4.16	4.16
W4@Au	3.92	4.12	4.11	4.12
W4~~@~~Au	4.10	4.15	4.15	4.16
W5@Au	3.99	4.12	4.10	4.09
W5~~@~~Au	4.10	4.15	4.15	4.16
W6@Au	3.81	4.13	4.13	4.13
W6~~@~~Au	4.06	4.16	4.15	4.15
W7@Au	3.87	4.13	4.12	4.09
W7~~@~~Au	4.08	4.16	4.14	4.15
W8@Au	4.03	4.13	4.13	4.03
W8~~@~~Au	4.10	4.15	4.14	4.15

Absolute values of the total Mulliken spin density at the four iron sites for the eight different AIMD walkers are calculated including (@) and excluding (~~@~~) the electronic effect of the gold substrate.

SUPPLEMENTARY NOTES

Supplementary Note 1

The general expression of the Mössbauer absorption cross-section in function of the energy ω for a quantum system described by a density matrix ρ connected to a set of eigenstates φ is[8]

$$\sigma(\omega) = \frac{2}{\Gamma_a} \Re\left\{\sum_\varphi \int_0^\infty e^{-pt} \langle\varphi|\rho A(t) A^\dagger(0)|\varphi\rangle \, dt\right\} \qquad (1)$$

where $\Re$ indicates the real part of the expression, $p = -i\omega + \Gamma_a/2$ and $A(t)$ represents the Hamiltonian describing the nucleus-radiation interaction. Moreover, Γ_a is the linewidth of the generic excited state. The cited expression contains a trace: this feature implies that any complete set of base states can be used to evaluate it.

In the present case φ can be chosen as the product of three independent states

$$|\varphi\rangle = |S, M_S\rangle \cdot |I, M_I\rangle \cdot |b\rangle$$

where $|S, M_S\rangle$ is the spin state of the molecular cluster, $|I, M_I\rangle = \prod_{i=1}^4 |I_i, M_{I_i}\rangle$ describes the nuclear spin state of the four iron ions of the cluster ($|I_i, M_{I_i}\rangle$ are the spin eigenfunctions of the ith nucleus) and $|b\rangle$ represents the thermal bath interacting with the spin state of the molecule. Moreover, $A(t) = \sum_{i=1}^4 A_i(t)$, where $A_i(t)$ is the Hamiltonian describing the interaction between the ith iron nucleus and the radiation. Consequently, the absorption cross-section can be rewritten as the sum of four terms depending on $A_i(t)$ and $|S, M_S\rangle \cdot |I_i, M_{I_i}\rangle \cdot |b\rangle$.

In addition, the spin levels of each iron nucleus are split by the local electric quadrupole tensors and by the hyperfine magnetic fields generated by the state $|S, M_S\rangle$ at each nuclear position; these last terms depend on the spin mean values $\langle s_z^i\rangle$,which are easily evaluated for each $|S, M_S\rangle$ eigenfunction.

Obviously $A_i(0)$ operates on the nuclear $|I, M_{I_i}\rangle$ states only and the identical property is also valid for the $A_i(t)$ Hamiltonian in the case of stationary cluster electronic states. Therefore, each iron nucleus will contribute to the absorption cross-section by the superposition of $2S + 1 = 11$ thermally mediated terms (each one due to the local hyperfine interactions generated by the generic electronic spin state).

In the non-stationary case, the time evolution operator depends also on the Hamiltonian terms that make the $|S, M_S\rangle$ states non-stationary and consequently $A_i(t)$ will also operate on the electronic and thermal bath parts of $|\varphi\rangle$. Therefore, the cross-section expression differs from the static one and will depend on the transition frequencies among the electronic $|S, M_S\rangle$ states. In the present case, $|S, M_S\rangle$ are not stationary states, both because they interact with a thermal bath and because of the presence of the E parameter in the spin Hamiltonian, which determines the so-called tunnelling of the magnetization. The spin-bath interaction determines a loss of coherence of the cluster eigenfunction and is described by introducing a Hamiltonian term of the form $H_{sb} = F_b Q_S(S_x, S_y, S_z)$. Taking into account only the linear terms of Q_S, the interaction Hamiltonian can be rewritten as $H_{sb} = S_x F_b$, where S_x induces transitions between electronic spin states with different M_S, while F_b operates on the thermal bath and can be expressed in terms of creation and annihilation operators, according to the particular spin-bath interaction. The E term of the spin Hamiltonian does not produce a loss of coherence of the

cluster spin state and can be described in terms of the transition frequencies between the otherwise degenerate states $\pm M_S$, as evaluated in ref. [9].

Supplementary Note 2

Under certain hypotheses each contribution to the absorption cross-section can be rewritten in the following way[8]

$$\sigma(\omega) = \frac{2}{\Gamma_a}\Re\{\int_0^\infty e^{-pt} Tr_{at}[\rho_{at}\langle A(t)\rangle_b A^\dagger(0)]\, dt\} \quad (2)$$

where, for the sake of simplicity, the "i" index, is omitted. Moreover, $\langle A(t)\rangle_b$ and Tr_{at} represent the average of the $A(t)$ operator over the thermal bath and the trace over the atomic states, respectively. The expression contains the matrix elements $A(p) = \int_0^\infty e^{-pt}\langle A(t)\rangle_b\, dt$ that can be demonstrated to be linear combinations of the matrix elements of $A(0)$.

Writing $A(p)$ as a vector in the space of transitions rather than as a matrix in the standard space of the states introduces a useful method to derive it. Using this framework, $A(p)$ can be obtained by applying the super-operator $G(p)^{-1} = [p - iH_{at}{}^x + R(p)]^{-1}$ to $A(0)$, where $H_{at}{}^x$ is the Liouville super-operator associated with the atomic Hamiltonian having $|S, M_S\rangle$ as eigenstates and $R(p)$ is the relaxation super-operator that reflects the dynamics of the quantum system.

Thus, evaluating the Mössbauer spectrum implies reconstructing $G(p)^{-1}$ through the computation of the right and left eigenvalues and eigenvectors of the non-Hermitian G operator. Shortly, the Mössbauer absorption cross-section is expressed by[8]

$$\sigma(\omega) = \frac{2}{\Gamma_a}\Re\left[\sum_\alpha \frac{\sum_{k,l} VRL_{k,l}^\alpha B_{l,k}}{-i\omega + \omega_\alpha}\right] \quad (3)$$

where the indices *k* and *l* stand for the various possible nuclear transitions of the static atomic system and α numerates the generic transition when the spin dynamics is considered. Moreover, $B_{l,k}$ depends on the matrix elements of the $A(0)$ operator; matrices $VRL_{k,l}^\alpha = VR_k^\alpha \times VL_l^\alpha$ are the tensor products of the right and left eigenvectors of G and, finally, ω_α are the eigenvalues of G.

First, we stress that the cross-section, as expressed by Supplementary Equation (3), does not have a Lorentzian shape. The $VRL_{k,l}^\alpha$ terms are complex numbers and, consequently, dispersive terms also appear in the cross-section. The imaginary parts of the eigenvalues ω_α define the central positions of both Lorentzian and dispersive terms, while the real parts of ω_α, which contain Γ_α and contributions of $R(p)$, determine the linewidth of Lorentzian and dispersive terms.

The dependence of the spectrum on the spin dynamics is due to the matrix elements $R_{i,j}$ among the states connected by the spin dynamics. However, a specific $R_{i,j}$ value does not identically affect all the lines of the spectrum due to the $|i\rangle$ and $|j\rangle$ electronic states. In fact, the key parameters to describe the effect of the spin dynamics on the lines are the ratios $R_{i,j}/|\omega_i - \langle\omega_{i,j}\rangle|$, where ω_i is the position of the generic line in the hyperfine fields generated by the electronic state $|i\rangle$ and $\langle\omega_{i,j}\rangle = \frac{\omega_i + e^{-\Delta/T}\omega_j}{1+e^{-\Delta/T}}$ is the mean value for the line position due to the hyperfine fields generated by the electronic states $|i\rangle$ and $|j\rangle$ differing by Δ in energy.

In our case, the spin dynamics is due to $\Delta M_S = \pm 1$ transitions with changes in the magnetic field value of the order of 10 T; consequently, all the differences $|\omega_i - \langle\omega_{i,j}\rangle|$ are smaller than 1 mm s^{-1}, while in the classical simple case of spin inversions the connected transitions are

located at opposite velocity values and consequently the maximum difference is of the order of 10 mm s^{-1}.

In spite of this important difference, to illustrate the main features of a spectrum in the presence of spin dynamics, we refer to the simple case of two degenerate electronic states generating opposite magnetic fields and the same electric field gradient (EFG). In this model the inversion of the electronic spin state is related to transitions between opposite M_S states within the S fundamental spin state.

In particular, Supplementary Figure 14 shows the evolution of the spectrum in the very simple case of a static quadrupole interaction and a magnetic one that stochastically changes its sign. In such case, transition (1), giving rise to the first line (from left) of the sextet, interacts with the sixth, transition (2) interacts with the fifth and finally the third interacts with the fourth. The key parameters describing the spectra are the three ratios $R/\Delta_{i,j}$, where R is the inversion rate between the $+M_S$ and $-M_S$ electronic states and $\Delta_{i,j} = |\omega_i - \omega_j|$, with ω_i and ω_j the nuclear frequencies calculated for the static situation.

For $R/\Delta_{i,j} \ll 1$, the main effect of the R value consists in an increase of the real part of the eigenvalues of G, while the absolute value of the imaginary part is only a little reduced with respect to the $R = 0$ case. Moreover, the real and imaginary parts of the right and left eigenvectors of G tend to be of comparable magnitude, giving rise to distortions of the line shapes that lose their Lorentzian trends, which is observed for $R = 0$.

For $R/\Delta_{i,j} = 1$, the real part of the eigenvalues of G is of the order of $\Delta_{i,j}$, while the imaginary part is equal to $(\omega_i + \omega_j)/2$. Consequently, the part of the cross-section shape of each couple of transitions is so large and unstructured to be hardly distinguishable from the spectrum baseline.

For $R/\Delta_{i,j} > 1$, the real part of the eigenvalues of G collapses to Γ_a, the imaginary part remains null and the lines tend to recover the original Lorentzian shapes, as R increases. Consequently, the growing of R produces the increase of a line centered at $(\omega_i + \omega_j)/2$ with a correlated lowering of the linewidth.

From these features the observable dynamic range can be partitioned into three regimes, which are referred to as slow, intermediate, and fast, respectively.

The slow dynamic regime (see panel a of Supplementary Fig. 14) is characterized by all $R/\Delta_{i,j}$ lower than 1, the intermediate one (see panel b of Supplementary Fig. 14) is characterized by R and $\Delta_{i,j}$ of the same order of magnitude, and finally the fast dynamic regime (see panel c of Supplementary Fig. 14) is characterized by all $R/\Delta_{i,j}$ much greater than 1.
Because $\Delta_{1,6} > \Delta_{2,5} > \Delta_{3,4}$, the contributions to the cross-section resulting from the three couples of transitions may not belong to the same dynamical range. This property permits to distinguish the spectral features arising from dynamical hyperfine interactions from those due to distributions of hyperfine magnetic or electrical interactions.
In the slow and intermediate dynamic regions, the linewidth of the internal lines is greater than those of the intermediate and external ones; moreover, for $\Delta_{3,4} < R < \Delta_{2,5}$ the spectrum shows five lines instead of six. Finally, for $R \sim \Delta_{1,6}$ the spectrum shape is composed by a moderately large peak superimposed to a very broad V shaped base (see panel b of Supplementary Fig. 14).

For fast dynamics, the spectrum appears markedly asymmetric, because of the collapse of the broad V shaped part. Finally, by increasing R, the asymmetry tends to disappear and the two lines recover an identical Lorentzian shape of natural linewidth.

In conclusion, the spectrum shape is very different from the one due to distributions of electrical hyperfine interactions (*i.e.* quadrupole interaction or isomer shift) or magnetic fields. Moreover, in the first case, all the lines of the spectrum are affected by identical enlargements; in the second case, the broadening of the external lines is expected to be around six times greater than that of the most internal ones. This factor is due to the difference between the Zeeman splitted excited and fundamental nuclear states. In particular, for the sixth line the energy value deriving from the separation between the levels splitted only by the magnetic interaction is proportional to

$$\left(\frac{3}{2}g_{ex} - \frac{1}{2}g_g\right)B_{hf}$$

where B_{hf} is the hyperfine magnetic field and g_{ex} and g_g are the nuclear g values of the excited and ground states, respectively. For the fourth line the relation is instead:

$$\left(-\frac{1}{2}g_{ex} - \frac{1}{2}g_g\right)B_{hf}$$

For ^{57}Fe the values of g_{ex} and g_g are -0.1031 and 0.1808, respectively. Therefore, the ratio between the sixth and fourth lines is close to six[10]. Considering that the spectra of the monolayer sample are characterized by the same broadening of all the absorption lines, this has been mainly ascribed to a distribution of electrical parameters.

It is also worth noting that a distribution of magnetic fields may be due to the thermal population of excited levels. In such case, the single characteristic contribution of each level will be always present; consequently, the temperature dependence of the spectrum is determined by the change in the relative weights of the contributions. This change may determine, upon increasing T, the appearance of more lines, as in Supplementary Fig. 15, or a broadening of the existing lines, if the values of the hyperfine fields, due to the various levels, are close to each other. However, in this last situation, the enlargements will be much greater for the external lines than for the intermediate and internal ones.

Then, we can conclude that distributions of hyperfine parameters will never give rise to spectra appearing as an intense single central line mounting on a broad V shaped base.

Supplementary Note 3

The spin levels of the ^{57}Fe nucleus are 2 for the fundamental state (I = 1/2) and 4 for the excited state (I = 3/2). In the present case S = 5 and the electronic states are 11, implying that the complete G matrix is of order $(2 \times 4) \times (11 \times 11) = 968$ and can be written as a series of $(11 \times 11)^2 = 14641$ squared blocks of order 8, corresponding to the different possible couples of transitions between the electronic states. Obviously, the diagonal blocks refer to transitions that do not affect the electronic spin states. Since $A(0)$ operates only on nuclear states, the cross-section depends on the only elements of $A(p)$ that are diagonal on the electronic part of $|\varphi\rangle$. Under reasonable approximations, this implies that only the G matrix elements referring to transitions that keep unaltered the spin state need to be considered. Therefore, the evaluation of the cross-section implies the diagonalization of a non-Hermitian matrix of order 88 composed by $(11 \times 11) = 121$ squared blocks with dimension 8.

In the case that $|S, M_S\rangle$ were stationary states, $R(p)$ would be zero and the only finite elements would be due to the Liouville super-operator ${H_{at}}^x$ and localized inside the 11 diagonal blocks; consequently, the cross-section relative to a certain iron nucleus would be the sum of 11 contributions with weights reflecting the thermal population of the electronic multiplet levels of the molecule. The non-stationarity of the $|S, M_S\rangle$ state, owing to the interaction with the thermal bath or the presence of the E term in the spin Hamiltonian, gives rise to finite terms in the non-diagonal blocks of the G matrix. For what concerns the spin-bath interaction, the contributions contain the Laplace transform of the correlation functions evaluated at the frequencies of the corresponding electronic transitions. These contributions, under certain hypotheses, can be written as in the formulae 27 and 28 of ref. [8].

The R matrix elements due to rhombic anisotropy (E) determine imaginary terms proportional to the transition frequencies among the otherwise degenerate $\pm M_S$ states, that are localized in blocks outside the previously mentioned 121 ones[9]; consequently, the portion of the G matrix to be diagonalized doubles in size. However, in simple cases, it is possible to show that the correct values for the $A(p)$ elements contributing to the cross-section can be obtained replacing those imaginary terms by real contributions equal to the square of the original ones and opportunely located inside the blocks referring to transitions that keep the spin state unaltered.

Considering that the main effect on the cross-section is due to the spin-bath interaction and the strong reduction in the computation time, the cross-section was evaluated using the mentioned substitution procedure. Furthermore, for the so-called sum rule, the sum with opposite sign of all the real elements situated on the corresponding row must be added to the generic term on the principal diagonal of the G matrix.

Supplementary Note 4

The matrix elements of R in the time domain are given by a sum of terms proportional to ${F_b}^2 = \langle n|F_b|n'\rangle\langle n'|F_b|n\rangle$ (n and n' are two bath states with energy separation ω_b), multiplied by $e^{-i\omega_{\mu,\nu}t}\, e^{i\omega_e t}e^{i\omega_b t}e^{-\frac{t}{\tau}}$, where the frequencies depend on the particular nuclear, electronic and bath transitions; moreover, τ is the lifetime of the quantum bath states. Consequently, in the energy domain, the matrix elements contain terms depending on the real part of $\left[-i\left(\omega-\omega_{\mu,\nu}+\omega_e+\omega_b\right)+\tau^{-1}+\Gamma_a\right]^{-1}$ that, in the White Noise Approximation (WNA)[8], becomes $\frac{\tau^{-1}}{(\omega_e+\omega_b)^2+\tau^{-2}}$ multiplied by an expression that depends on the spin-bath interaction.

In general, the particular expression of the generic matrix element of R will depend on the actual interaction scheme for the spin-bath interaction, on the density of bath states, on the lifetime τ and finally on the energy difference between the electronic states.

If $\tau^{-1} \gg \omega_e$, the energy differences among the spin states can be disregarded, and the matrix elements of R are independent of the particular spin transition (see ref. [8]).

When the above condition cannot be applied, the R elements may depend on the frequencies of the spin transitions, but in most cases the dependence is expected to be not very strong and the necessity to reduce the number of free parameters may induce to consider the elements of R as approximately independent on the particular electronic transition.To illustrate the subject some notes follow.

Let us consider two simple dependencies of F_b on the bath states creation and annihilation operators:

- a linear dependence, as for a direct process,
  $F_b \propto \sum c_n\left(a_n + a_n^\dagger\right)$
- a quadratic dependence, as for indirect processes,
  $F_b \propto \sum c_{n,m}\left(a_n a_m^\dagger + a_n^\dagger a_m\right)$.

In the first case, the matrix element $\langle n|F_b|n'\rangle\langle n'|F_b|n\rangle$ for a transition with gain in energy by the electronic spin system (*i.e.* ω_e positive) will contain the matrix element of $a_n^\dagger a_n$ and $\omega_b$will be negative. The matrix element for the inverse transition will be characterized by opposite frequencies and described by the matrix element of $a_n a_n^\dagger$. Therefore, the two matrix elements of R will depend on the mean value on the bath states of $f_e = \frac{\tau^{-1}}{(\omega_e-\omega_b)^2+\tau^{-2}}\frac{1}{e^{\frac{\omega_b}{T}}-1}$ and $\frac{\tau^{-1}}{(\omega_e-\omega_b)^2+\tau^{-2}}\frac{e^{\frac{\omega_b}{T}}}{e^{\frac{\omega_b}{T}}-1} = f_e e^{\frac{\omega_b}{T}}$, respectively.

In the second case (quadratic dependence), the matrix elements will depend on the mean value of terms $\frac{\tau^{-1}}{(\omega_e-\omega_{b1}+\omega_{b2})^2+\tau^{-2}}\frac{e^{\frac{\omega_{b1}}{T}}}{\left(e^{\frac{\omega_{b1}}{T}}-1\right)\left(e^{\frac{\omega_{b2}}{T}}-1\right)} = f_e$ and

$\frac{\tau^{-1}}{(\omega_e-\omega_{b2}+\omega_{b1})^2+\tau^{-2}}e^{\frac{\omega_{b1}}{T}}\frac{e^{\frac{\omega_{b2}-\omega_{b1}}{T}}}{\left(e^{\frac{\omega_{b1}}{T}}-1\right)\left(e^{\frac{\omega_{b2}}{T}}-1\right)} = f_e\ e^{\frac{\omega_{b2}-\omega_{b1}}{T}}$with $\omega_{b1} > \omega_{b2}$ respectively.

To obtain the matrix elements of R, the previous expressions must be multiplied by the density of bath states, the coupling coefficients c_n or $c_{n,m}$, integrated on the bath state frequencies, and finally multiplied by the matrix elements of S_X^2.
Therefore, considering that only the energies of the electronic states are known, it appears reasonable to introduce some approximations in order to minimize the number of free parameters.

While the $\tau^{-1} \gg \omega_e$ approximation cancels out the dependence of R on ω_e, the presence of the term $\frac{\tau^{-1}}{(\omega_e-\omega_b)^2+\tau^{-2}}$ suggests that the opposite approximation ($\tau^{-1} \ll \omega_e$) will give rise to the major dependence of the R matrix elements on the electronic energy differences. In the following, we discuss the simplest dependencies obtained in this last approximation; other possible dependencies are reported in ref. [10,11].
Therefore, the matrix elements of R for both direct and indirect processes may be approximately written as proportional to $\tau \cdot f_e(T)$ and $\tau \cdot e^{\frac{\omega_e}{T}} f_e(T)$, for transitions where the electronic spin system gains and loses energy, respectively.

For direct processes, $R(T) \propto \rho(\omega_e)\frac{1}{e^{\frac{\omega_e}{T}}-1}$, then the matrix elements of $R(T)$ depend on the particular electronic transition. Moreover, they depend exponentially on the temperature as $e^{-\frac{\omega_e}{T}}$ for $T \ll \omega_e$, while, for $T \gg \omega_e$, the elements will depend linearly on the temperature, that is $\frac{T}{\omega_e}$.
For second order processes, $R(T)$ will depend on the quantities $\rho(\omega_b)\rho(\omega_b - \omega_e)\frac{e^{\frac{\omega_b-\omega_e}{T}}}{\left(e^{\frac{\omega_b-\omega_e}{T}}-1\right)\left(e^{\frac{\omega_b}{T}}-1\right)}$ and their temperature dependencies will be described by $e^{-\frac{\omega_b}{T}}$ at low T, independently of ω_e, and $\frac{T^2}{\omega_b(\omega_b-\omega_e)}$ at high T (weakly dependent on ω_e), respectively.
Consequently, an independence of the $R(T)$ on the electronic transitions (apart from the dependence of the matrix elements of S_X^2) can be introduced in order to reduce the number of free parameters.

In conclusion, apart from the case of a direct process and large values for the lifetime of the bath states ($F_b \propto \sum c_n\left(a_n + a_n^\dagger\right)$; $\tau^{-1} \ll \omega_e$), the approximation of considering $R(T)$ for transitions with a gain in energy of the electronic spin system as independent on the particular electronic frequency seems to be reasonable. Therefore, this can be usefully used to fit the spectra with the warning of reconsidering the procedure, if the results suggest the presence of direct processes.

Supplementary Note 5

A brief description of the Supplementary Figures is here reported.

To facilitate the comparison between the spectra of the dropcast and monolayer samples at 2.2 K, the two spectra are superimposed in Supplementary Fig. 1. In the monolayer sample the external lines change their shape and shift toward the centre of the spectrum with respect to the dropcast sample.

In Supplementary Fig. 2-4, the spectra of dropcast and monolayer samples at 2.2 K, 7.0 K and 11 K are reported in the same figures. The *x* axes of the monolayer spectra are scaled with respect to those of the dropcast sample (by a factor equal to the ratio of the experimentally determined hyperfine fields) in order to directly compare the linewidth and intensity of the absorption lines for the two samples. Apart from the S/N ratio, the main difference is the linewidth, which is on average larger for the monolayer sample. Therefore, we can conclude that the spin dynamics is not modified, *i.e.* the same dynamic regimes hold for the two samples at each temperature.

In order to show the accuracy of the fitting procedure, in Supplementary Fig. 5-6 the spectra measured at 2.2 and 40 K on the dropcast sample are presented together with the corresponding fit and residuals.

Supplementary Fig. 7 reports the magnetic hyperfine fields extracted from the spectra collected at 2.2 K and plotted as a function of $\left|\langle s_z^i \rangle\right|$, *i.e.* the absolute value of the average local spin components along the *z* axis. These values were estimated from the spin Hamiltonian (Equation 1 in the main text) with the parameters obtained from *ab initio* calculations[12] on the optimized structure in the bulk phase and on the 8 walkers of the *ab initio* molecular dynamics (AIMD) modellization of the monolayer. The assignment of the observed hyperfine fields to specific iron ions was straightforward for the dropcast sample, considering the distinct Mössbauer parameters of the central ion and the occurrence of two inequivalent peripheral ions in a 1:2 ratio. The experimental hyperfine fields are in good agreement with the so-called $22\langle s_z \rangle$ rule for the Fermi contact field.[13] Turning now to the monolayer sample, the $|\langle s_z^i \rangle|$ value calculated for each iron ion (i) was averaged over the 8 walkers. The association to the experimental hyperfine fields was based on the expected proportionality of the latter to the averaged $|\langle s_z^i \rangle|$.

In Supplementary Fig. 8, the absorption cross-section obtained by fitting the spectrum of the dropcast sample at 11 K is compared with the cross-section simulated for T = 15 K (or correspondingly a 27% reduced $|D|$ value) while leaving the other parameters unchanged, and with the cross-section simulated at T = 11 K for a higher transition rate (in particular, that expected at 15 K). It is evident that while an increased temperature changes the intensity of the lines, an increased transition rate between different states causes changes in lineshapes and shifts the lines towards the centre of the spectrum. The simulated behaviour is encountered in the dropcast sample (see Figure 2a in the manuscript): for $T \leq 11$ K, the thermal evolution of the spectra is dominated by the population of excited spin states, while at higher temperatures it is dominated by the increased transition rates.

In order to show the accuracy of the fitting procedure, in Supplementary Fig. 9-10 the spectra measured at 2.2 and 11 K on the monolayer sample are presented together with the corresponding fit and residuals.

In Supplementary Fig. 11, the Mössbauer spectrum of the monolayer sample at 2.2 K and the corresponding fit are compared with a simulation of the same sample, in which (as texture parameter) the anisotropy axes of the SMM molecules were assumed to lie at about 35° from the substrate normal, as previously found[4]. Such a preferential orientation determines a redistribution of line intensities and yields an asymmetrical pattern that does not correspond to the experimental data. Therefore, this simulation confirms that Mössbauer spectra of the monolayer sample are correctly fitted considering a random orientation of molecules.

Supplementary Fig. 15 shows two simulations of the high temperature cross-sections without considering the interaction with the thermal bath and setting either $E = 0$ or $E = 0.003$ K (the value extracted from the spectrum of the dropcast sample at 2.2 K). The two curves differ only in the range -4 mm/s ÷ +4 mm/s, indicating that the contributions of $M_S = \pm5$, ±4 and ±3 doublets are practically independent of E. On the contrary the remaining contributions strongly depend on the spin Hamiltonian terms giving rise to the magnetization tunnelling effect.

In Supplementary Fig. 16, a modified version of Figure 3a in the manuscript is presented with the cross-sections of the three contributions in logarithmic scale, so as to better evidence for each contribution the peaks associated to the thermal population of the cluster spin states. The main peaks of each contribution arise from the $M_S = \pm5$ doublet, while the secondary peaks arise from $M_S = \pm4$ and ±3. Moreover, the small central peak is due to the E term in the spin Hamiltonian.

SUPPLEMENTARY METHODS

Synthetic Methods

95%+ enriched ^{57}Fe was supplied by Wissenschaftliche Elektronik GmbH, Germany. Diethyl ether from a freshly-opened can was pretreated with $CaCl_2$ overnight, filtered, and distilled from its sodium benzophenone ketyl solution under dinitrogen prior to use. Methanol and 1,2-dimethoxyethane were refluxed over $Mg(OMe)_2$ and NaH, respectively, and distilled with protection against moisture before use. NaOMe (3.139 M in methanol) was prepared by careful addition of Na metal (1.804 g, cut into small pieces), to methanol (25 mL) under dinitrogen. 7-(Acetylthio)-2,2-bis(hydroxymethyl)heptan-1-ol (H_3L) was synthesized as described elsewhere[1]. All remaining chemicals were reagent grade and were used as supplied, unless otherwise noted. Single-crystal X-ray diffraction measurements were carried out at room temperature using a Bruker-Nonius X8APEX four-circle diffractometer with Mo-K_α radiation. Electrospray Ionization mass spectrometry (ESI-MS) in positive ion mode was performed on an Agilent Technologies 6310A Ion Trap LC-MS(n) spectrometer by direct infusion of solutions in 2-propanol:CH_2Cl_2 3:1 v/v (~0.5 mg/mL). The synthesis of [$^{57}Fe_4(L)_2(dpm)_6$] complex followed established literature methods with only minor modifications[2].

$^{57}FeCl_3$. ^{57}Fe metal (73.04 mg, 1.283 mmol) was introduced in a Kjeldahl flask and dissolved in a mixture of concentrated HCl (0.41 mL) and concentrated HNO_3 (0.14 mL). The solution was treated with 7 drops of concentrated HCl, the original volume restored by evaporation using a heat gun and the procedure repeated two more times. The solution was finally concentrated by heating until incipient precipitation and any solid traces redissolved by adding the minimum required amount of concentrated HCl. Under fast dinitrogen flux, the solution was cautiously treated with an excess of freshly-distilled thionyl chloride (evolution of sulfur dioxide and hydrogen chloride occurred) and gently heated to remove all volatiles[3]. The black lustrous solid ($^{57}FeCl_3$) so obtained was dissolved in methanol (10 mL) to give a 0.1283 M solution.

[$^{57}Fe_2(OMe)_2(dpm)_4$]. Three-fourths of the $^{57}FeCl_3$ solution (7.50 mL, 0.962 mmol) were diluted to 12 mL with methanol and a solution of Hdpm (0.355 g, 1.93 mmol) and NaOMe (1.13 mL, 3.55 mmol) in methanol (3 mL) was added dropwise with stirring over about 10 min. The reaction mixture was stirred overnight and the copious yellow-orange precipitate of [$^{57}Fe_2(OMe)_2(dpm)_4$] filtered on a G3 sintered glass frit, washed with 3 mL of methanol and thoroughly dried in vacuum (0.393 g, 89.9%).

[$^{57}Fe_4(OMe)_6(dpm)_6$]. [$^{57}Fe_2(OMe)_2(dpm)_4$] (0.393 g, 0.432 mmol) was suspended in Et_2O:MeOH 2:1 v/v (36 mL) and stirred for 10 minutes. $^{57}FeCl_3$ solution in methanol (0.1283 M, 2.25 mL, 0.289 mmol) was added dropwise with stirring to give a clear, dark-red solution which was stirred for 10 minutes and treated dropwise with NaOMe (3.139 M in methanol, 0.275 mL, 0.863 mmol) under vigorous stirring. Upon the addition, the solution turned to yellow-orange and a yellow precipitate appeared (the final pH was close to neutrality). After stirring for further 15 minutes, Et_2O (98 mL) was added to the mixture, which was stirred for 2 h and left undisturbed overnight. The precipitated NaCl was filtered off on a G4 sintered glass frit and washed with a few mL of Et_2O. Vapour diffusion of methanol (250 mL) into the combined yellow-orange organic phases gave [$^{57}Fe_4(OMe)_6(dpm)_6$] as yellow rods in 2 weeks

(vapour diffusions on such large volumes of solutions were conveniently carried out in a large desiccator; the product solution was contained in a wide-neck Erlenmeyer flask or in a crystallizing dish placed on top of a ceramic baffle plate and the incoming solvent was poured directly into the desiccator). The product was isolated by filtration, washed with the external Et_2O:MeOH diffusion mixture and dried in vacuum. Few orange-red crystals of $[^{57}Fe_2(OMe)_2(dpm)_4]$ were easily removed manually (0.205 g, 47%).

$[^{57}Fe_4(L)_2(dpm)_6]$. $[^{57}Fe_4(OMe)_6(dpm)_6]$ (40.7 mg, 26.9 μmol) was dissolved in Et_2O (10 mL) and treated with solid H_3L (19.3 mg, 77.1 μmol) under stirring. After complete dissolution of the ligand, the clear yellow-orange solution was slowly evaporated to dryness over a liquid paraffin trap in a desiccator. The yellow-orange solid so obtained was extensively washed with methanol on a G3 sintered glass frit, vacuum dried and then redissolved in the minimum amount of 1,2-dimethoxyethane. Slow complete evaporation of the solvent over a liquid paraffin trap in a desiccator gave $[^{57}Fe_4(L)_2(dpm)_6]$ as yellow lustrous thin plates mixed with rare yellow-orange prisms. The product was extensively washed with methanol until colourless washings and dried in vacuum (29 mg, 59%). The two crystal phases were identified as known polymorphs of $[^{57}Fe_4(L)_2(dpm)_6]$ by single crystal X-ray diffraction at room temperature[4]. ESI-MS: *m*/*z* 1844.7 ($[M+Na]^+$, 100%), 1860.6 ($[M+K]^+$, 29%), see Supplementary Figure 12 for the mass spectra.

Mössbauer Experiments

Mössbauer spectra of the Fe_4 samples were measured at the Nuclear Resonance Beamline[5] ID18 of the European Synchrotron Radiation Facility (ESRF), taking advantage of the Synchrotron Mössbauer Source (SMS) [6,7]. This device achieves unique performances by filtering the broad synchrotron light (~15 meV) coming out from the high-resolution monochromator into an ^{57}Fe-resonant (~14.4 keV) narrow single line, using pure nuclear reflections of an iron borate ($^{57}FeBO_3$) crystal, heated to the vicinity of the Néel temperature. The details and the technical aspects of the setup are described elsewhere[6,7].

It is useful to stress here the key differences and advantages of SMS with respect to a conventional Mössbauer setup. In contrast to common radioactive sources, the radiation coming from the SMS is a needle-like collimated beam with small (~ mm) size, which is further focused to spot sizes of micrometer lateral dimensions[7]. Furthermore, it is fully polarized (with the electric field vector lying in the laboratory vertical plane) and fully recoilless (it does not contain any background).

The energy spectrum of the radiation to a first approximation can be described by a squared Lorentzian distribution[7], for which the full-width-at-half-maximum (FWHM) and intensity values derive from the temperature of the iron borate crystal. For this reason, a compromise between spectral resolution and intensity of the SMS source must be chosen. The line used in the present study had a FWHM approximately three times larger than for a radioactive source and an intensity of about 1.5×10^4 photons per second. To estimate the FWHM of the line, Mössbauer spectra of a single-line absorber ($K_2Mg^{57}Fe(CN)_6$) of known thickness were collected before and after each Mössbauer measurement on the samples and fitted using the source FWHM value as free parameter. Therefore, the FWHM of SMS radiation used in the fitting procedure of each sample spectrum was evaluated as the average between the FWHMs of the two corresponding single-line spectra. Moreover, these single-line spectra enabled the evaluation of the isomer shift of the SMS radiation with respect to the radiation utilized in a conventional Mössbauer setup: the SMS line resulted to be centred at 0.709 mm s^{-1} with respect to conventional α-Fe.

Mössbauer spectra were recorded by collecting the radiation reflected at grazing incidence by the sample surface. A grazing angle $\theta = 0.1°$ was chosen for both samples, after measuring their reflectivity as a function of the angle of incidence. This value of the incidence angle is larger than the critical angle of total external reflection from the molecular monolayer (low atomic number), but smaller than the critical angle of reflection from the gold substrate (high atomic number). Consequently, the grazing incidence reflection occurred from the surface of the substrate, whereas the layer of deposited molecules produced absorption lines.

In the case of small grazing angles, the spectra could be treated as those obtained in a standard transmission geometry setup. However, in grazing incidence geometry the effective thickness of the sample was multiplied by the factor $\frac{2}{sin\theta}$. This thickness amplification, which was of the order of 1100 in our experimental conditions ($\theta \sim 0.1°$), was key for a successful application of SMS to molecular monolayers, which would otherwise be impossible to investigate with a conventional Mössbauer setup.

For both samples, the Mössbauer spectra were recorded as function of the temperature and without applying any external magnetic field.

To quantify the contribution of Fe impurities in beryllium collimating lenses used in the beamline, an "empty-can" Mössbauer spectrum (*i.e.* a spectrum with no mounted sample) was also collected (Supplementary Figure 13). This spectrum evidences an intense absorption at zero velocity and significant baseline distortions. To correct the measured Mössbauer spectra for these effects, the fit of the "empty-can" Mössbauer spectrum was subtracted from the experimental spectra.

SUPPLEMENTARY REFERENCES

1. Tancini, E. *et al.* On-surface magnetometry: The evaluation of superexchange coupling constants in surface-wired single-molecule magnets. *Chem. Eur. J.* **19,** 16902–16905 (2013).
2. Accorsi, S. *et al.* Tuning anisotropy barriers in a family of tetrairon(III) single-molecule magnets with an S = 5 ground state. *J. Am. Chem. Soc.* **128,** 4742–4755 (2006).
3. Pray, A. R., Heitmiller, R. F., Strycker, S., Aftandilian, V. D., Muniyappan, T., Choudhury, D. & Tamres, M. Anhydrous Metal Chlorides. *Inorg. Synth.* **28,** 321–323 (1990).
4. Mannini, M. *et al.* Quantum tunnelling of the magnetization in a monolayer of oriented single-molecule magnets. *Nature* **468,** 417–421 (2010).
5. Rüffer, R. & Chumakov, A. I. Nuclear Resonance Beamline at ESRF. *Hyperfine Interact.* **97–98,** 589–604 (1996).
6. Smirnov, G. V, van Bürck, U., Chumakov, A. I., Baron, A. Q. R. & Rüffer, R. Synchrotron Mössbauer source. *Phys. Rev. B* **55,** 5811–5815 (1997).
7. Potapkin, V. *et al.* The 57Fe Synchrotron Mössbauer Source at the ESRF. *J. Synchrotron Radiat.* **19,** 559–569 (2012).
8. Spina, G. & Cianchi, L. in *NMR-MRI, μSR and Mössbauer Spectroscopies in Molecular Magnets* 249–275 (Springer Milan, 2007).
9. Cianchi, L., Del Giallo, F., Spina, G., Reiff, W. & Caneschi, A. Spin dynamics study of magnetic molecular clusters by means of Mossbauer spectroscopy. *Phys. Rev. B* **65,** 64415 (2002).
10. Cianchi L., Moretti P., Mancini M., Spina G. Mossbauer spectra in paramagnetic relaxing systems. *Reports Prog. Phys.* **49,** 1243–1291 (1986).
11. Gutlich, P., Eckhard, B. & Trautwein, A. X. *Mössbauer Spectroscopy and Transition Metal Chemistry*. **49,** (Springer-Verlag, 2011).
12. Lunghi, A., Iannuzzi, M., Sessoli, R. & Totti, F. Single molecule magnets grafted on gold: magnetic properties from ab initio molecular dynamics. *J. Mater. Chem. C* **3,** 7294–7304 (2015).
13. Greenwood, N. N. & Gibb, T. C. *Mössbauer Spectroscopy*. (Springer Netherlands, 1971).
14. Redman, J. E. *Isotope Distribution Calculator,* Cardiff University, UK, 2005, http://www.kombyonyx.com/isotopes/ (accessed October 2017).
